# Biochemistry and physiology of voltage-gated calcium channel trafficking: a target for gabapentinoid drugs

**DOI:** 10.1098/rsob.250013

**Published:** 2025-07-16

**Authors:** Annette C. Dolphin

**Affiliations:** ^1^ Neuroscience Physiology and Pharmacology, University College London, London, England, UK

**Keywords:** voltage-gated, calcium, channels, trafficking, target, drugs

## Abstract

Voltage-gated calcium channels are multi-pass transmembrane proteins that have key features that are essential for their correct function. For example, the concerted movement of the voltage sensor domains in response to depolarization is required to ensure the channels open over a particular range of voltages, essential for the physiological function of each of the 10 different mammalian calcium channels. Furthermore, their selectivity filter is required to maintain the Ca^2+^ selectivity of these channels. The auxiliary subunits, α_2_δ and β, play important roles in aiding the channels to fold, traffic and function correctly. Of therapeutic importance, the α_2_δ subunits are a drug target of the gabapentinoid α_2_δ ligands, whose mechanism of action is now better understood. However, much is still unknown about how these calcium channels reach and are retained in specific locations, and how these processes are modified by various forms of modulation and synaptic plasticity.

**Figure:**
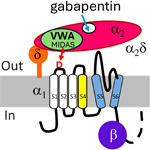

## 1. Introduction

Most ion channels are gated in some way, meaning their opening is controlled, for example, by factors such as an alteration in transmembrane (TM) voltage, and/or a change in intracellular factors, including cyclic AMP, ATP or Ca^2+^. Other ion channels are directly gated by extracellular ligands, particularly neurotransmitters. This ability to be gated is essential, both to maintain ionic gradients between the exterior and interior of cells, or within organelles, and also to respond specifically to particular physiological stimuli, including metabolic changes or neuronal activity. Voltage-gated calcium channels (Ca_V_) are gated by depolarization, and are exquisitely Ca^2+^-selective [1]. Particular Ca_V_ channels are also modulated directly or indirectly by intracellular Ca^2+^ [2], and by second messengers, including G proteins [3] and other factors including cyclic AMP-dependent phosphorylation [4,5].

## 2. Calcium channel structure and classification

**Figure 1.**
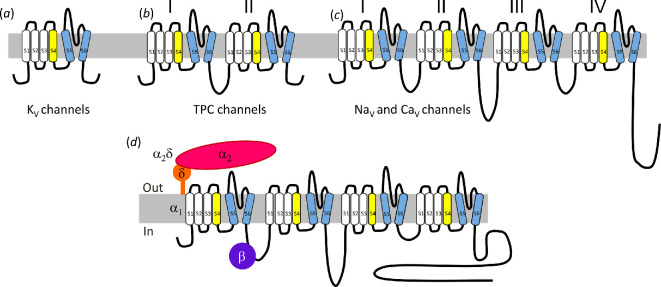
The topology of voltage-gated ion channels. The basic topology for (a) K_V_ channels (single 6 TM domain), (b) TPC channels (two 6 TM domains), (c) Na_V_ and Ca_V_ channels (four 6 TM domains). (d) The α_2_δ and β subunit interact with Ca_V_1 and Ca_V_2 α1 subunits to form the Ca_V_ channel complex.

Voltage-gated ion channels (VGICs) respond to a change in voltage through their voltage-sensor domains. Except in particular cases, such as hyperpolarization-activated channels ( see [6]), VGICs are activated by depolarization, often in addition to other ligands. The genes for these channels were initially cloned either from tissues in which they were strongly expressed, using ligands to follow their purification, or taking advantage of naturally occurring genetic mutations. Cloning studies indicated that K_V_ channels, Na_V_ α subunits and Ca_V_ α1 subunits are all membrane proteins with an intracellular N-terminus [7–10]. The sensor for a change in TM voltage is found in the voltage sensor domain, which is a module of four TM segments, with intracellular N- and C-termini, in which the fourth TM α-helix has multiple positively charged amino acids, that move outwards on depolarization (see [11]). This domain is attached to a two TM segment termed the pore domain, with an extracellular re-entrant pore (P) loop [12]. With respect to voltage-gated K^+^ channels, the voltage-sensor plus pore-domain forms a discrete subunit (figure 1a), four of which combine to form the channel complex, and this allows for multiple combinations of isoforms [13]. In endosomal two-pore channels (TPCs), two double subunits combine to form a channel [14,15] (figure 1b), whereas in eukaryotic voltage-gated Na^+^ channels, and voltage-gated Ca^2+^ (VGCCs), the four domains are concatenated to produce a single polypeptide [12] (figure 1c). With the availability of structures for most members of the Ca_V_ channel family, it is found that each pore domain is domain-swapped, being associated with the neighbouring voltage-sensor domain, rather than its own [16].

When the Ca_V_ channel complex was first purified from skeletal muscle t tubules, it was shown to contain five protein bands, termed α1 (approx. 170 kDa), α2 (approx. 150 kDa), β (approx. 52 kDa), δ (approx. 17−25 kDa) and γ (approx. 32 kDa) [17]. The α2 and δ polypeptides were then found to form a single disulfide-bonded protein [18].

For the Ca_V_ family, there are now known to be 10 mammalian VGCC α1 subunit genes, named *CACNA1A-I*, comprising four Ca_v_1 channels, three Ca_V_2 channels and three Ca_V_3 channels [19]. These Ca_V_ α1 pore-forming subunits all have 24 TM domains, some of which are not typical hydrophobic α-helices and represent particular challenges to their correct folding in the endoplasmic reticulum (ER). In part to promote correct folding, the Ca_V_1 and Ca_V_2 channels are all associated with auxiliary β and α_2_δ subunits, whereas the Ca_V_3 channels are not associated with obligate auxiliary subunits [20–22] (figure 1d). Although a γ subunit (γ_1_) is associated with the skeletal muscle Ca_V_1.1 (α1S) [16], this is not the case for the other Ca_V_1 or Ca_V_2 channels. Indeed, other members of the γ family of proteins were subsequently found to associate with glutamate receptors [23].

VGCCs are highly Ca^2+^ selective and a Ca^2+^ ion is present in the pore in many structures [1,16]. This selectivity is conferred by the structure of the re-entrant pore loops, whose correct organization is an important requirement for function. These pore loops each have a key negatively charged amino acid (glutamate in Ca_V_1 and Ca_V_2), which is essential for their Ca^2+^ selectivity and permeation [24,25]. Although pore-mutant channels have been used in some studies as non-permeant ‘dead’ channels [26,27], more recently, it was found that following mutation of these key glutamate residues, the channels do not traffic correctly, despite the presence of their auxiliary subunits [28]. This result indicates that the correct formation of the selectivity filter is likely to be essential for the structural integrity of the channels, and it emphasizes that trafficking and function are intertwined.

## 3. Ca_V_ β subunit binding and effect on proteasomal degradation

Ca_V_ β subunits are encoded by four mammalian genes with multiple splice variants [29]. They are crucially important for the correct folding, forward trafficking from the ER and function of all except Ca_V_3 channels. They bind to the I–II linker of the Ca_V_1 and Ca_V_2 channels (figure 1d), to a sequence called the alpha interaction domain (AID) [30]. Structural studies show the AID α-helix binds tightly to a groove in the guanylate kinase domain of the Ca_V_β subunits [31–33]. In the presence of Ca_V_ β, the AID region forms an extended α-helical structure in the α1 subunits [31–33]. Mutation of a key tryptophan in the AID sequence of Ca_V_2.2 dramatically decreases Ca_V_2.2 current density [34]. Interestingly, membrane localization of the β2 splice variant, β2a, by virtue of its dual N-terminal palmitoylation which independently mediates membrane association, mitigates this reduction [34]. This interaction of Ca_V_ β with the α1 I–II linker allows the Ca_V_ channels to avoid polyubiquitination and ER-associated proteasomal degradation (ERAD) [35,36]. There are multiple lysines in the I–II linker which can be ubiquitinated, and poly-ubiquitination of the Ca_V_2.2 I–II linker is prevented by Ca_V_ β binding [37]. In agreement with this, mutation of the lysine residues in the I–II loop of full-length Ca_V_2.2 channel was found to protect the channel from degradation [37].

Ca_V_ β subunits all increase Ca_V_ current density [29], in part by hyperpolarizing the voltage-dependence of activation [29], but also because they augment cell-surface expression of all Ca_V_1 and Ca_V_2 channels, where it has been investigated [30,38,39].

## 4. Ca_V_ α_2_δ subunit biosynthesis and domain structure

Ca_V_ α_2_δ subunits consist of two polypeptides (α_2_ and δ) linked by multiple disulfide bonds. Both are encoded by a single gene, *CACNA2D* [18], of which there are four isoforms *CACNA2D1-4* [40–42], each with multiple splice variants [40,43].

**Figure 2.**
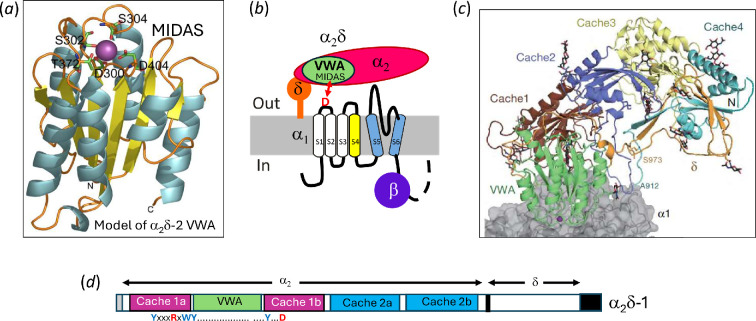
Domain structure of α_2_δ. (a) Model of the VWA domain in α_2_δ-2 shows the five MIDAS residues that co-ordinate a divalent cation (taken from [45]). (b) The first domain of Ca_V_2.2 shows the D in extracellular loop I which co-ordinates with the VWA domain MIDAS motif to complete the divalent cation co-ordination site. (c) α_2_δ-1 structure (reproduced from [16]). (d) Organization of α_2_δ-1 domains, shows the two polypeptides α_2_ and δ, and the positions of the VWA and double Cache domains. The grey region at the N-terminus represents the signal peptide, and the black region at the C-terminus represents the GPI anchor signal which is cleaved off. Beneath is shown the motif responsible for the universal amino acid binding site (adapted from [46]). (c) is reproduced with permission from Nature.

The *CACNA2D* genes all encode a pre-protein with an N-terminal signal sequence and a C-terminal hydrophobic region, initially suggesting they are type I TM proteins [18,40,44]. They contain several key domains, including a von Willebrand factor-A (VWA) domain (figure 2a–c), which is key to enhancement of Ca_V_ function by α_2_δ [16,47]. VWA domains are widely distributed extracellular protein–protein interaction domains, that interact in a divalent cation-dependent manner through a metal ion-dependent adhesion site (MIDAS) motif [48] (figure 2a). In addition, four Cache domains [49] are present in α_2_δ proteins [16] (figure 2c,d). These have recently been redefined as two double Cache domains, which are also present in all bacterial chemotactic receptors and chemotransducers that bind nutrient amino acids (figure 2d). In α_2_δ-1 and α_2_δ-2, the first of these double Cache domains contains the amino acid-binding site [46,50,51] (see §12–14 on gabapentinoids).

An α_2_δ-like protein, Cachd1, which is a TM protein, also contains Cache domains. It produces small enhancement effects on Ca_V_2.2 currents, which exactly parallel an increase in trafficking [52] and it also increases T-type currents [53]. However, a key function of Cachd1 is in the Wnt signalling pathway [54].

## 5. The role of the endoplasmic reticulum membrane complex complex in biogenesis and trafficking of the Ca_V_ complex

As described above, Ca_V_ α1 subunits are membrane proteins with an intracellular N-terminus [8,9]. It is important to note that the first extracellular loop of the α1 subunit (which will be within the ER during biogenesis) interacts with the VWA domain of the α_2_δ subunits. This occurs through its MIDAS motif associating with a conserved aspartate (D) (figure 2b,c) in the first extracellular loop of α1, to allow the MIDAS motif to co-ordinate a divalent cation [16,52]. It is possible that this interaction serves to stabilize the nascent α1 topology during biogenesis.

It is well known that fixing the orientation of the first TM segment of a membrane protein is critical for determining the correct topology of all subsequent TM α helices [55]. The ER membrane complex (EMC) is a complex of nine proteins associated with the ER membrane which has been shown to be involved in the biogenesis of multiple TM proteins including GPCRs and ion channels [56]. Its primary role is the insertion of the first TM segment of TM proteins, whereas the Sec61 complex is essential for insertion of the second TM α helix [57]. The EMC may also have further chaperone roles with respect to membrane proteins [56].

VGCCs are more complex than many TM proteins having 24 TM domains and four P loops. The structure of EMC in a complex with Ca_V_1.2/β has recently been determined; in which the interaction between the EMC and the Ca_V_1.2 α1 subunit involves an interaction between the first and second TMs of Domain I with the TM domain of EMC1. Other EMC proteins in the complex also interact with the Ca_V_ β subunit [58]. Intriguingly, in this study, the α_2_δ interaction with Ca_V_1.2/β was found to be mutually exclusive with the EMC complex [58]. In the EMC-bound conformation, multiple luminal parts of EMC1 occupy the position that would be occupied by α_2_δ binding. The EMC might thus be considered a ‘holdase’ retaining the immature calcium channel in the ER until it interacts with α_2_δ and attains a more mature structure.

## 6. Post-translational modification of α_2_δ

Although initial studies indicated that the C-terminus of α_2_δ-1 had a TM domain, closer inspection of the characteristics of the C-terminal sequences of all the α_2_δ subunits, as well as further experimentation indicated that these proteins are all glycosyl-phosphatidylinositol (GPI)-anchored extracellular proteins, and this modification is essential for their function [45]. GPI anchors are glycolipid moieties that are pre-formed in the ER and then attached in the ER to an ω-amino acid in a motif near the C-terminus of the protein, with the remaining C-terminal sequence being cleaved off [59,60]. GPI-anchored proteins are found to be associated with cholesterol-rich lipid raft membrane fractions, which influence their trafficking pathways and their membrane distribution [61].

According to well-established biochemical pathways of membrane protein synthesis [62,63], following the synthesis of α_2_δ proteins, their folding, intra-protein disulfide bond formation, GPI anchor addition and initial N-glycosylation, all occur in the ER [45,64]. Glycosylation of proteins is a complex process [65] involving the addition of pre-formed glycan units, which in the case of N-glycosylation are linked to asparagine, and this occurs in the ER lumen. For α_2_δ-1 proteins there are 16 N-glycosylation sites [66]. These glycans are then further modified and trimmed in the Golgi apparatus, to form mature glycosylated α_2_δ proteins.

## 7. Importance of proteolytic cleavage of α_2_δ for its function

The mature glycosylated form of α_2_δ proteins is also the proteolytically cleaved form [64]. Our evidence indicates that proteolytic cleavage of α_2_δ proteins is mainly associated with the Golgi apparatus [67]. However, when a thrombin cleavage site is substituted for the endogenous cleavage site experimentally, proteolytic cleavage of α_2_δ-1 cannot occur in the Golgi, but can be induced to occur on the cell surface by external application of thrombin [67]. Our studies indicate that before cleavage of α_2_δ, its interaction with the α1 subunit is weaker and mutant α_2_δ proteins that cannot be cleaved do not enhance calcium channel currents [67]. One key protease involved in this cleavage process is a member of the disintegrin and metalloprotease (ADAM) family (ADAM17), whose knockout or pharmacological inhibition reduces proteolytic cleavage of α_2_δ-1 [68].

In our studies, α_2_δ proteins cause up to a 12-fold increase in maximum conductance for Ca_V_2.1 and Ca_V_2.2 currents [52,69,70], whereas the effect on cell-surface expression is often a smaller fold increase [38,67]. It is possible that the greater increase in Ca_V_ currents is because of the additional channel activation step mediated by proteolytic cleavage of α_2_δ [68].

## 8. Effect of α_2_δ on forward trafficking versus endocytosis of Ca_V_ channels

Where it has been studied, the trafficking of Ca_V_1 and Ca_V_2 VGCCs is increased by α_2_δ proteins, both *in vitro* [38] and *in vivo* [71,72], although for the stably anchored skeletal muscle Ca_V_1.1 channels, present in tetrads in the plasma membrane of *t* tubules, it has been reported that α_2_δ does not affect the amount of channels at the cell surface [73]. To account for these differences, it is possible that Ca_V_2 channels are more affected by α_2_δ subunits because they are more mobile in the plasma membrane, particularly in presynaptic terminals where rapid plasticity is required [74,75].

**Figure 3.**
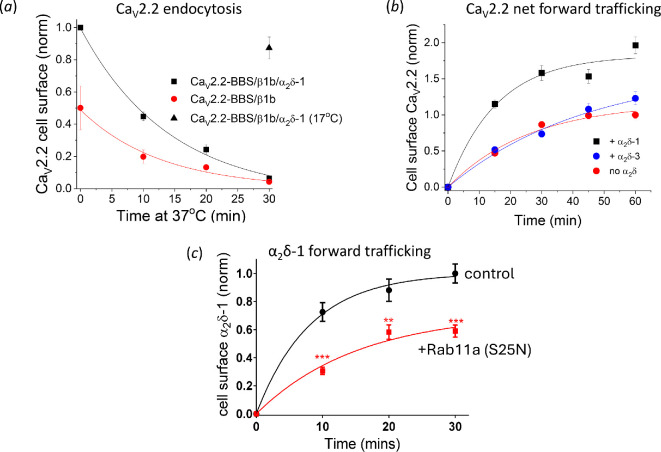
The effect of α_2_δ proteins on forward trafficking and endocytosis. (a) To examine endocytosis, Ca_V_2.2-BBS was measured on the cell surface after incubation at 37°C for 0−30 min of cells expressing the subunits shown. The time constant τ for the plotted exponential fits was 14.1 min for Ca_V_2.2-BBS/β1b/α_2_δ-1-HA (black line) and 12.8 min for Ca_V_2.2-BBS/β1b (red line). (Adapted from fig. 3f in [38], in which experimental details may be found.) (b) To examine net forward trafficking, Ca_V_2.2-BBS was measured on the cell surface after 0−60 min incubation at 37°C, for cells expressing Ca_V_2.2/β1b and either α_2_δ-1 (black), α_2_δ-3 (blue) or no α_2_δ (red). τ = 15.6 min (+α_2_δ-1), 26.1 min (no α_2_δ), and 44.9 min (+α_2_δ-3). (Adapted from fig. 5b in [76], in which experimental details may be found.) (c) Net forward α_2_δ-1-BBS trafficking showing time course for cell-surface α_2_δ-1-BBS, either alone (black circles) or with Rab11a(S25N) (red squares). Single exponential fits have τ = 8.1 min for α_2_δ-1 control and 19.5 min for α_2_δ-1 + Rab11 a (S25N). Statistical significance versus control: ***p < 0.0001; ** *p* = 0.0018. (Adapted from fig. 6bb in [76], in which experimental details may be found.)

The presence or absence of α_2_δ-1 was not found to influence the rate of internalization of Ca_V_2.2 from the plasma membrane [38] (figure 3a), but strongly influenced the forward trafficking by about threefold over a period of an hour [76] (figure 3b). Forward trafficking was also increased by α_2_δ-2 but surprisingly it was not affected by α_2_δ-3 over the same time course [76] (figure 3b). In the same study, using a dominant-negative Rab11(S25N) construct, which is known to interfere with trafficking through recycling endosomes [77], it was found that cell-surface expression and forward trafficking of Ca_V_2.2 was strongly inhibited by Rab11(S25N) in the presence of α_2_δ-1, as was the trafficking of α_2_δ-1 itself (figure 3c). In contrast, cell-surface expression of Ca_V_2.2 in the presence of α_2_δ-3 was not influenced by Rab11(S25N), indicating that α_2_δ-3 is unable to contribute to this process of recycling of calcium channel subunits back to the cell surface [76]. Interestingly the amino acid binding site in α_2_δ-3 is partially occluded [50].

## 9. The α_2_δ subunit is more than a trafficking chaperone

The α_2_δ subunits result in a marked increase in currents in the case of all Ca_V_ calcium channels studied, for α_2_δ-1 [9,78], α_2_δ-2 [47,79], α_2_δ-3 [41] and α_2_δ-4 [42]. Furthermore, α_2_δ proteins also shift steady-state inactivation to more hyperpolarized potentials and produce an increase in inactivation rates [41,47,80–82], as well as other biophysical effects on the channels, which are all indicative of the subunits remaining associated with the channels on the cell surface.

## 10. Mouse and human mutations in *CACNA2D* genes

There are several naturally occurring mutations in mouse *Cacna2d2* that result in disruption of the full-length α_2_δ-2 protein, including ducky (*du, du^2J^*) [79,83] and *entla* [84]. These recessive mutations produce a severe phenotype of cerebellar ataxia and seizures. Furthermore, related to the fact that α_2_δ-2 is strongly expressed in cerebellar Purkinje cells, there is a decrease in Purkinje neuron calcium currents and Purkinje neuron firing is also reduced in *du^2J^* mice [79,85]. Human biallelic loss-of-function mutations in *CACNA2D2* also result in severe neurodevelopmental delay and epilepsy [86–88].

For *Cacna2d1,* there is a reduction of cardiac, chromaffin cell and dorsal root ganglion neuron calcium currents in α_2_δ-1 knockout mice [82,89,90], and increased susceptibility for diabetes in some mouse strains [91]. The α_2_δ-1 knockout mice also have an abnormal pain phenotype, showing reduced mechanical and cold sensitivity, and delayed development of mechanical hyperalgesia following neuropathic injury [89]. In humans, biallelic loss-of-function mutations in *CACNA2D1* also result in severe epileptic encephalopathy and neurodevelopmental delay, and also loss of pain sensibility [92]. These findings concerning pain are likely to relate to the up-regulation of α_2_δ-1 following peripheral nerve injury, and the mechanism of action of gabapentinoid α_2_δ ligands (§14).

## 11. Trafficking and function of Ca_V_2 channels in presynaptic terminals

Most presynaptic terminals rely mainly on Ca_V_2.1 and Ca_V_2.2 channels for the entry of Ca^2+^ necessary to trigger synaptic release (except in the inner ear and the retina [93]), and these channels are clustered close to release sites, which themselves are aligned to post-synaptic receptors [94–102].

There is still a lot that is unclear about how Ca_V_2 channels are trafficked to, and retained in, active zones where they mediate transmitter release [93]. In some studies, Ca_V_2.1 channel clustering increases with development [103]; however, in a GABAergic terminal (cerebellar basket cell synapse) that is dependent on Ca_V_2.1, it was found that these channels formed nanoclusters throughout development, while docked vesicles became more clustered during development [99].

Multiple proteins, including Rim, Rim-binding protein and Munc-13, have been implicated in anchoring Ca_V_2 channels, directly or indirectly, in the presynaptic active zone, in close proximity to docked vesicles [94,103,104]. Interestingly, Ca_V_2.2 channels, together with α_2_δ-1 and α_2_δ-2, and also synaptotagmin-11, have been identified as part of the GABA_B_ receptor proteome [105]. Furthermore, it has recently been demonstrated that synaptotagmin-11 binds to the GABA_B_ receptor auxiliary subunit KCTD16, and to Ca_V_2.2 channels, and recruits these channels to post-Golgi trafficking vesicles [106], at least in part for axonal transport. It has also been found that presynaptic active zone expansion is likely to be delivered by the fusion of dense core vesicles containing various essential components [107].

**Figure 4.**
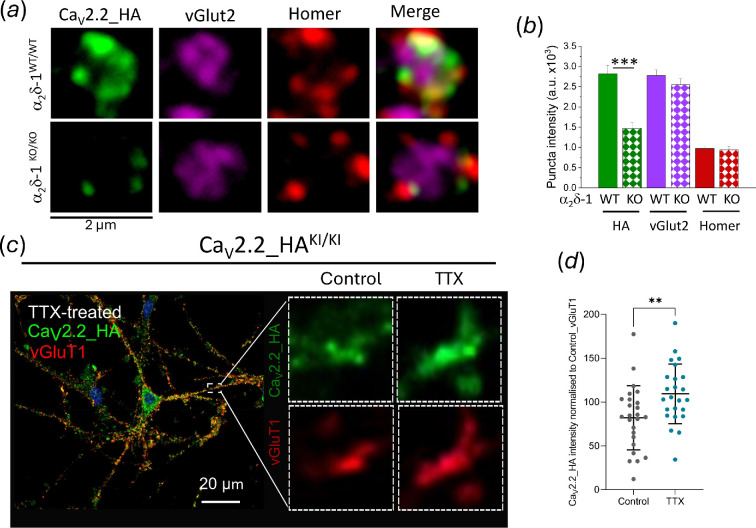
Presynaptic localization of Ca_V_2.2. (a) Airyscan images (2 × 2 μm) of individual rosette clusters of Ca_V_2.2_HA puncta taken from superficial dorsal horn of Ca_V_2.2_HA^KI/KI^ α2δ-1^WT/WT^ (top row) and Ca_V_2.2_HA^KI/KI^ α2δ-1^KO/KO^ (bottom row) mouse sections, showing co-localization with pre- and post-synaptic markers. L–R: Ca_V_2.2 (HA, green), vesicular glutamate transporter-2 (vGlut2 Ab, magenta), Homer (Ab, red) and merged image. (b) Puncta intensity for Ca_V_2.2_HA^KI/KI^ α2δ-1^WT/WT^ (solid bars) and Ca_V_2.2_HA^KI/KI^ α2δ-1^KO/KO^ (chequered bars) for Ca_V_2.2_HA (HA Ab, green bars), vGlut2 (magenta bars), and Homer (red bars). ***p = 0.0014 (Student’s *t*-test). ((a) and (b) Adapted from fig. 6c,d in [72], in which experimental details may be found.) (c) Left: Airyscan image of a TTX-treated cultured hippocampal neuron from Ca_V_2.2_HA^KI/KI^ mouse at day-*in-vitro* 21, stained with anti-HA Ab (green) and anti-vGluT1 Ab (red) to identify presynaptic terminals. Magnification ×63, scale bar 20 µm. Right: 3 × 3 µm subset images from areas such as white box in (c). Control boutons (left panels) and TTX-treated (right panels) with Ca_V_2.2_HA in green and vGluT1 in red. (d) Increased Ca_V_2.2_HA intensity in TTX-treated neurons compared with control neurons as mean ± s.d. N for control neurons = 25 and for TTX-treated = 24, from three independent experiments. For each neuron, up to 75 ROIs were selected. Data were normalized to the averaged control value. Two-tailed unpaired *t*‐test, *p* = 0.006. ((c) and (d) Adapted from fig. 4g,i, in [75], in which experimental details may be found.)

In relation to these findings, it has been well studied that GABA_B_ receptors are involved in presynaptic inhibition [108], and inhibit Ca_V_2 channels, including in primary afferent neurons [109]. We observed using hemagglutinin (HA)-tagged Ca_V_2.2 knock-in mice that knockout of a_2_δ-1 almost abolished the localization of Ca_V_2.2_HA in primary afferent terminals in the superficial dorsal horn of the spinal cord, which is likely to be an effect on trafficking [71,72] (figure 4a,b). Furthermore, homeostatic plasticity, induced by tetrodotoxin to reduce neuronal activity, results in an increase in presynaptic Ca_V_2.2, determined both by imaging and by increased presynaptic Ca^2+^ flux [75] (figure 4c,d). Long-term potentiation and depression are also likely to involve concomitant pre- and post-synaptic changes to be most efficient [107,110,111].

Functional interaction of Ca_V_2.2 with mature α_2_δ proteins is important for their presynaptic function and their localization [112], and impaired Ca_V_2.2 function [28] or impaired interaction with α_2_δ [52], reciprocally affects their trafficking in neurons. There are also several key determinants in the Ca_V_2.2 C-terminus, which are important for their trafficking [113,114]. The C-terminus of Ca_V_2.1 is also important for its presynaptic function [115].

## 12. Localization and function of Ca_V_ channels in dendrites

Although essential presynaptic roles of Ca_V_ channels have been widely studied, these channels also have very important post-synaptic functions to propagate and amplify post-synaptic signals received by dendritic spines, in conjunction with ligand-gated ion channels, such as NMDA receptors. The spatial and temporal integration of both depolarization and elevation of post-synaptic Ca^2+^ are important for signal propagation [116]. The relevance of L-type channels (particularly Ca_V_1.2) in dendrites has been shown in a number of studies [117–120], but other Ca_V_ channels also play key roles, including Ca_V_2 and Ca_V_3 subtypes [121–124].

How entry of Ca^2+^ into dendrites mediates changes in gene expression in the nucleus (the process of excitation–transcription coupling) has also been widely studied, with multiple second messenger systems being implicated [125–127]. In this regard, a remarkable repeating organization of dendritic ER contacts with the plasma membrane has recently been revealed, in which multiple Ca_V_ subtypes (including Ca_V_2.1, Ca_V_2.2 and Ca_V_1.2) are clustered at regularly spaced ER-plasma membrane junctions along the dendrites [128]. Junctophilin-3 is required for this clustering of Ca_V_ channels. These junctions form hotspots of Ca^2+^ entry and amplification of the signal by activation of Ca^2+^-dependent Ca^2+^ release through ryanodine receptors. This repeating structure might therefore amplify signals from individual dendritic spines, propagating the signal along dendrites [128].

## 13. Development of the gabapentinoid α_2_δ ligands

Gabapentin was developed as a GABA analogue, and found to have anti-epileptic efficacy [129], although its mechanism of action remained unclear. It was then found to bind to a protein in the brain that, when isolated, was identified as α_2_δ-1 [130,131].

**Figure 5.**
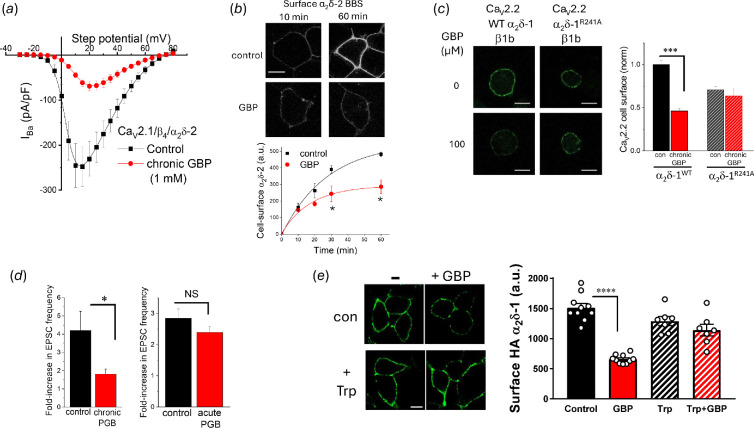
The effect of gabapentinoids on Ca_V_ currents and Ca_V_ trafficking. (a) Effect of 1 mM gabapentin (GBP, red) applied for 40 h compared with control (black) on Ca_V_ current-voltage relationship for channels formed by Ca_V_2.1/β4/α_2_δ-2 expressed in tsA-201 cells. (Adapted from fig. 1A in [70], in which experimental details may be found.) (b) Effect of GBP on forward trafficking of α_2_δ-2 from the recycling endosome compartment, isolated using brefeldin A. Top: representative images, scale bar: 20 µm refers to all images; bottom: time course for mean cell-surface expression (control: black; GBP: red). The effect is statistically significant at 30 and 60 min (*p < 0.05). (Adapted from fig. 5B,D in [135], in which experimental details may be found.) (c) Effect of 100 µM GBP for 24 h on cell-surface expression of Ca_V_2.2, together with β1b and α_2_δ-1 or α_2_δ-1^R241A^. Left: representative images in presence of α_2_δ-1 (left panel) or α_2_δ-1^R241A^ (right panel). Scale bars: 10 µm. Right: bar chart of mean data for the four conditions indicated. The effect of GBP is statistically significant for α_2_δ-1, but not for α_2_δ-1^R241A^ (*p* < 0.001). (Adapted from fig. 4A,B in [38], in which experimental details may be found.) (d) Capsaicin-evoked fold-increase in ESPC frequency recorded from spinal cord neurons co-cultured with DRGs. Left: fold-increase in ESPC frequency when cultures were incubated for 40−48 h with 100 μM pregabalin (PGB) compared with control (*p < 0.05, Student’s *t-*test). Right: lack of effect of acute PGB (1 mM) on fold-increase in enhancement of EPSC, compared with control. (Adapted from fig. 4D,H in [134], in which experimental details may be found.) (e) Left: representative images of tsA−201 cells expressing HA-tagged α_2_δ-1 in the absence or presence of 1 mM GBP and absence (upper panel) or presence (lower panel) of 1 mM L-tryptophan (Trp). Scale bar: 10 µm refers to all images. Right: bar chart showing mean data (bars) and individual experiments (open circles) for control (black),+GBP (red), Trp (black hatched) and Trp+GBP (red hatched). Statistical significance of control versus GBP in the absence of Trp is *p* < 0.0001. (Adapted from fig. 3 in [50], in which experimental details may be found.)

When it was applied acutely in dorsal root ganglion (DRG) neurons, or cells transfected with calcium channel subunits, gabapentin was then found either to have no effect [70] or variable small inhibitory effects [132] on calcium channel currents. In contrast, gabapentin applied for 17−40 h had a greater effect, producing about 50% inhibition of Ca_V_2.2 currents at 100 µM–1 mM [70,133] (figure 5a). This inhibition by chronically applied gabapentin was only observed when the auxiliary subunits α_2_δ-1 or α_2_δ-2 were included (not α_2_δ-3 which does not bind gabapentin), and it was absent if a mutation of the third arginine (R) in a triple arginine motif (present in both α_2_δ-1 and α_2_δ-2) was made in these subunits [70]. These mutations had previously been shown to markedly reduce gabapentin-binding affinity [69,131,135].

## 14. Mechanism of action of the gabapentinoid α_2_δ ligands

Our initial evidence suggested that gabapentin reduced cell-surface expression of α_2_δ-2 and Ca_V_2.1 [70,133]. Subsequently, we extended these studies using a bungarotoxin-binding site (BBS)-tagged α_2_δ-2, and we were able to isolate an intracellular site of action of gabapentin with α_2_δ proteins [136]. We found that gabapentin reduced the forward trafficking of α_2_δ subunits back to the cell surface from recycling endosomes by nearly 50% over an hour [136] (figure 5b). The recycling endosome compartment was isolated by the use of brefeldin A to block trafficking through the Golgi apparatus, and the effect of gabapentin was also blocked by the dominant-negative Rab11 (S25N), which blocks movement of proteins from recycling endosome to the cell surface [136]. In a further advance, we utilized a Ca_V_2.2 construct, with a BBS tag in an extracellular loop where function was not affected, and we found that gabapentin (100 µM for 24 h) reduced cell-surface expression of Ca_V_2.2 in the presence of wild-type α_2_δ-1, but not in the presence of the α_2_δ-1 mutant in which the third R in its triple arginine motif is changed to A [38] (figure 5c).

*In vivo,* neuropathic injury to primary afferent sensory nerves results in elevation of α_2_δ-1 mRNA expression in the injured DRG cell bodies [137] and increased α_2_δ-1 protein expression in both DRG cell bodies [138] and their terminals in the dorsal horn [139]. In this study, we found that chronic dosing of the gabapentinoid drug pregabalin for 9 days in rats subjected to spinal nerve ligation reduced the elevation of α_2_δ-1 in the dorsal horn of the spinal cord, following this neuropathic injury [139].

In a subsequent study, we recorded excitatory post-synaptic currents (EPSCs) from dorsal horn neurons in DRG-dorsal horn co-cultures, and selectively activated EPSCs from presynaptic DRG terminals using capsaicin (figure 5d). We found that chronic pregabalin (100 µM applied for 40−48 h), reduced the frequency of these EPSCs [134]. In contrast, even a higher concentration of pregabalin (1 mM), applied acutely, had no effect [134] (figure 5d). Since there may be a relatively rapid turnover of synaptic calcium channels, which contribute to synaptic plasticity [75], it is likely that gabapentinoid drugs act more rapidly on the trafficking of calcium channels in some synaptic terminals, particularly where α_2_δ-1 is elevated following neuropathic insult [140,141].

Pregabalin, when applied chronically, has also been found to reduce cell-surface expression of α_2_δ-1 and Ca_V_1.2 in cerebral arterial smooth muscle cells and reduce myogenic tone [142]. Whether this contributes to its therapeutic action or side-effects is unclear.

## 15. Gabapentinoids bind to a universal amino acid-binding site on α_2_δ

The question of why there is a gabapentinoid drug-binding site on the α_2_δ proteins, α_2_δ-1 and α_2_δ-2, was in part solved recently [46]. As discussed above, bacterial chemoreceptors, which sense nutrient amino acids contain double cache domains, in which the amino acid-binding site is situated. In eukaryotic genomes, these double cache domains are only found in α_2_δ proteins and in Cachd1 [46]. It appears that this double cache domain has been employed by α_2_δ proteins for both structural and functional purposes. The universal amino acid binding site is characterized by a structural motif in the first double cache domain in α_2_δ-1 and α_2_δ-2 (figure 2d) [46,50]. Structural studies have confirmed the binding of gabapentin to this site [51]. Of note, mutation of the R in this conserved motif in both α_2_δ-1 and α_2_δ-2 impaired the ability of the mutant α_2_δ subunit to enhance Ca_V_ channel function and trafficking, implying amino acid binding to this site might be important for optimal function [70,136,143]. Furthermore, a deleterious point mutation in human *CACNA2D1* is in a conserved residue in the environment of this amino acid-binding site [92].

These and other findings indicate that the presence of an endogenous amino acid in this binding site may be important for the normal function of the α_2_δ proteins, as suggested previously [70]. Indeed, the apparent affinity of α_2_δ-1 for gabapentin increases with its purification, implying the displacement of an endogenous ligand, and ^3^H-leucine competes with gabapentin [144]. In agreement with this, two amino acids (tryptophan and phenylalanine) that are predicted to bind to this site with high affinity prevent the inhibitory effect of gabapentin on α_2_δ-1 trafficking [50] (figure 5e). Furthermore, structural studies indicate the presence of the abundant amino acid leucine in the universal amino acid-binding site of α_2_δ-1 [58].

## 16. Do α_2_δ proteins traffic with other proteins?

Although Ca_V_2 channel trafficking is strongly enhanced by α_2_δ subunits, these α_2_δ proteins can reach the cell surface alone [38,136], opening the possibility that they interact on the cell surface with other proteins. Thrombospondins were identified as a family of extracellular interacting proteins, specifically with α_2_δ-1 [145], although several studies could not reproduce key aspects of this potential interaction [146,147]. Furthermore, neurexins were proposed to interact extracellularly with α_2_δ proteins to mediate retrograde synaptic inhibition [148], but they were subsequently found not to form stable direct complexes [149]. In other studies, it has been found that presynaptic expression or deletion of different α_2_δ proteins differentially affects synapse formation in neuronal cultures, which may in part be independent of effects on calcium channel function [150–152], implying interaction with other ligands.

Since the EMC and α_2_δ are mutually exclusive in their interaction with Ca_V_ α1/β complexes, it is possible that the EMC, which interacts with many TM proteins [153], might also be displaced by α_2_δ with respect to these proteins. Indeed, α_2_δ-1 proteins have been observed to interact with the ionotropic glutamate-gated ion channels, NMDA receptors [154] and AMPA receptors [155], although they have not been observed in the proteomes of these receptors [156]. Interestingly, the interaction with these ion channels was reported to be via the C-terminus of α_2_δ-1, although this is cleaved off post-translationally, during GPI anchor addition [45], and therefore it is likely that any such interaction is restricted to the ER. This and other potential interactions have been reviewed in detail recently [157].

## 17. Conclusion

Ca_V_ channels present particular challenges with respect to their biogenesis and trafficking. They have charged residues in their voltage sensor TM domains, and they have four re-entrant loops that form the extracellular turret and lining of the pore with the selectivity filter. The correct operation of the voltage sensor domain is required to ensure the channels open over a particular range of voltages, essential for their physiological function, and the selectivity filter is required to maintain the Ca^2+^ selectivity of these channels. Auxiliary α_2_δ and β subunits play important roles in aiding the channels to fold and traffic correctly, and α_2_δ subunits represent a fortuitously identified drug target for the gabapentinoid drugs, whose mechanism of action has been elucidated in recent years. However, much is still unknown about how the different Ca_V_ channels reach and are retained in specific locations, and how this is modulated in various forms of synaptic plasticity and by the gabapentinoid α_2_δ ligands.

## Data Availability

This article has no additional data.
